# Perspectives on health policy dialogue: definition, perceived importance and coordination

**DOI:** 10.1186/s12913-016-1451-1

**Published:** 2016-07-18

**Authors:** Juliet Nabyonga-Orem, Kevin Ousman, Yolanda Estrelli, Adzodo K. M. Rene, Zina Yakouba, Mesfin Gebrikidane, Drave Mamoud, Aku Kwamie

**Affiliations:** 1 Health Systems and Services Cluster, World Health Organization Regional Office for Africa, Cite de Djoue, BP 06 Brazzaville, Republic of Congo; 2 Health Systems and Services, World Health Organization Cabo Verde Country Office, PO Box 266, Praia, Cabo Verde; 3 Health Systems and Services, World Health Organization Guinea Country Office, PO Box Boîte postale 817, Conakry, Guinea; 4 Health Systems and Services, World Health Organization Chad Country Office, PO Box Boîte postale 152, N’Djamena, Chad; 5 Health Systems and Services, World Health Organization Liberia Country Office, PO Box 316, Monrovia, Liberia; 6 Health Systems and Services Cluster, World Health Organization Togo Country Office, PO Box BP 1504, Lomé, Togo; 7 University of Ghana, School of Public Health, P.O. Box LG 13, Accra, Ghana

**Keywords:** Policy dialogue, Coordination, Stakeholders

## Abstract

**Background:**

Countries in the World Health Organization African Region have witnessed an increase in global health initiatives in the recent past. Although these have provided opportunities for expanding coverage of health interventions; their poor alignment with the countries’ priorities and weak coordination, are among the challenges that have affected their impact. A well-coordinated health policy dialogue provides an opportunity to address these challenges, but calls for common understanding among stakeholders of what policy dialogue entails. This paper seeks to assess stakeholders’ understanding and perceived importance of health policy dialogue and of policy dialogue coordination.

**Methods:**

This was a cross-sectional descriptive study using qualitative methods. Interviews were conducted with 90 key informants from the national and sub-national levels in Lusophone Cabo Verde, Francophone Chad, Guinea and Togo, and Anglophone Liberia using an open-ended interview guide. The interviews were transcribed verbatim, coded and then put through inductive thematic content analysis using QRS software Version 10.

**Results:**

There were variations in the definition of policy dialogue that were not necessarily linked to the linguistic leaning of respondents’ countries or whether the dialogue took place at the national or sub-national level. The definitions were grouped into five categories based on whether they had an outcome, operational, process, forum or platform, or interactive and evidence-sharing orientation. The stakeholders highlighted multiple benefits of policy dialogue including ensuring stakeholder participation, improving stakeholder harmonisation and alignment, supporting implementation of health policies, fostering continued institutional learning, providing a guiding framework and facilitating stakeholder analysis.

**Conclusion:**

Policy dialogue offers the opportunity to improve stakeholder participation in policy development and promote aid effectiveness. However, conceptual clarity is needed to ensure pursuance of common objectives. While it is clear that stakeholder involvement is an important component of policy dialogue, numbers must be manageable for meaningful dialogue. Ownership and coordination of the policy dialogue are important aspects of the process, and building the institutional capacity of the ministry of health requires a comprehensive approach as opposed to strengthening selected departments within it. Likewise, capacity for policy dialogue needs to be built at the sub-national level, alongside improving the bottom-up approach in policy processes.

## Background

The countries in the World Health Organization (WHO) African Region have seen an increase in global health initiatives and new partnerships for health in the recent past [1]. Although these have provided opportunities for expanding coverage of health interventions, they have come with challenges, including their poor alignment with the countries’ priorities and weak coordination, among others [1]. In addition, health development calls for engagement of line ministries outside the health sector, as well as non-state actors, to support various aspects in implementing the health sector strategic plan [2]. Engagement of the health-related sectors and non-state actors in health development is ineffective still. For example, the governments’ challenge of increasing funding allocation to health has been related to communication gaps and misunderstanding between the ministries of health and finance [3]. Policy dialogue creates an opportunity for the different stakeholders to deliberate, forge relationships, generate common solutions and move towards mutually agreed objectives [4]. The principles of aid effectiveness highlight the importance of aligning actors’ interventions and resources to nationally owned plans [5], and a well-coordinated health policy dialogue provides an opportunity to realise that objective. Policy dialogue involves people from different interest groups sitting together to focus on an issue in which they have mutual interest although not necessarily common perspectives [6].

Although policy dialogue is fairly common in various sectors in many countries, conceptual clarity of the term is yet to be realised [4]. Policy dialogue in health has taken various definitions, depending on the context and policy issue under consideration. For example, the Romanow Commission on the future of health care in Canada adopted the term “ChoiceWork dialogue”, which they defined as dialogues that engage members of the public as stakeholders in the health sector on important issues before decisions are made, with the goal of achieving consensus [7]. Lavis et al. [8] use the term “deliberative dialogue”, defined as a group process that can help to integrate and interpret scientific and contextual evidence for the purpose of informing health policy development. Hartz-Karp [9] on the other hand uses the term “deliberative democracy”, which he sees as a mechanism that strengthens the voices of citizens in governance. The Paris Declaration and Accra Agenda for Action urge governments – in the case of health the ministry of health – to take the leading role in the health agenda as well as health policy dialogue [5].

To attain the goals of generating common solutions and moving towards mutually agreed objectives, stakeholders must have a common understanding of what policy dialogue entails. This points to the need to assess stakeholders’ perceived understanding as well as the importance they attribute to health policy dialogue in different settings. Some scholars have argued that the differences in understanding of the concept are based on linguistic considerations and the level of the health system where the policy dialogue takes place [4]. In view of this, Liberia, a post-conflict and anglophone country that is also recovering from the 2014 Ebola disease outbreak, Cabo Verde, a lusophone country, and the francophone countries of Guinea – also recovering from the Ebola outbreak – Chad and Togo were selected for a comparative analysis aimed to identify the differences and similarities in their understanding of what policy dialogue entails, to help guide the efforts in strengthening health policy dialogue and contribute towards improving aid effectiveness. These countries were chosen based on the fact that they had been implementing a health policy dialogue programme for their national health policies and strategic plans for universal health coverage (UHC) since 2012 under the UHC Partnership of the European Union, the Government of Luxembourg and WHO.

The objectives of this paper are to assess stakeholders’ understanding and perception of the concept of policy dialogue, its importance, and responsibility for its coordination and to evaluate the similarities and differences on these elements from national and sub-national level perspectives, as well as among francophone, anglophone and lusophone countries in Africa. The various themes that emerge from this study will guide future planning of health policy dialogue processes in the WHO African Region and low income countries elsewhere. In addition, the study is expected to make a contribution to the body of empirical evidence on policy dialogue, especially in low and middle income countries, which is scanty at the moment.

## Methods

### Study design

This was a cross-sectional, descriptive study using qualitative methods. The study aimed to assess stakeholders’ understanding of what policy dialogue is and its perceived importance, as well as its coordination. Using an interview guide, we collected data from 90 key informants in Cabo Verde, Chad, Guinea, Liberia and Togo at both the national and sub-national levels. Data collection was undertaken June–August 2015.

### Selection of respondents

At the national level, the initial step was to hold discussions with WHO country office and ministry of health (MoH) technical officers responsible for convening policy meetings, who identified the key agencies involved. At the sub-national level, the first step was to meet with the head of the district health office, who identified the MoH officers and agencies involved in the policy dialogue process. Within the identified agencies, the key informants were purposively selected based on their participation in the policy dialogue process and seniority [10]. Additional key informants were identified through snowballing, until descriptive saturation was achieved [11]. All the informants were invited to participate in the interviews by phone, and 98 % percent of them agreed and were interviewed (see Table 1).

**Table 1:** Key informants by country

	Cabo Verde	Chad	Guinea	Liberia	Togo
National level					
Ministry of health	5	6	15	4	8
Donor agencies	1	4	6	3	1
Civil society	6	0	3	3	4
Sub-national level					
Ministry of health	0	4	3	1	4
Donor agencies	0	0	0	0	0
Civil society	2	0	1	2	4
Total	14	14	28	13	21

### Data collection

Data were collected using an interview guide consisting of open-ended questions that explored the respondents’ understanding of what policy dialogue is, their perceived importance of policy dialogue and their view on who coordinated the policy dialogue process at the national and sub-national levels. In each participating country data were collected by a team of independent researchers, who were all experts in qualitative research and were knowledgeable on health policy and systems research. Interviews, which lasted 45 min on average, were conducted in English in Liberia, French in Chad, Guinea and Togo, and Portuguese in Cabo Verde and were all audiotaped.

### Data analysis

All the interview responses were transcribed verbatim and later translated into English by a specialised team at the WHO Regional Office for Africa. The translated responses were reviewed by English speaking technical officers in the WHO country offices to ensure that the meaning was preserved, and were read by the research team to have an appreciation of the emerging issues vis-à-vis the research questions. They were then coded using QRS Nvivo Software Version 10. Using inductive manifest and thematic content analysis, the research team assessed how the respondents defined policy dialogue, their perception of its importance and their view on its coordination (see Table 2 for an example). An attempt was made to determine if there were differences in responses among the countries, as well as between the national and sub-national levels. The themes identified were reviewed by the team for consensus and, where interpretation differed, the raw data were revisited and discussed to harmonise the views.

**Table 2:** In your view, what is the importance of health policy dialogue?

Response	Category 1 (manifest)	Category 2 (manifest)	Theme
The PD was invaluable when we were reviewing the progress the ministry had made over the period. We held a general health conference where we had to do an analysis comparing the achievements of the previous period against the targets we had set. We convened stakeholders not only from the MoH but also health colleagues from the county level, partners and donors. We reviewed progress and this galvanised the sector.	Brings key stakeholders together to review health sector performance.	Ensures participation of stakeholders in assessing health sector performance.	Ensures stakeholder participation in planning and monitoring.
Having the annual review and bringing together policy-makers to plan and assess the ministry's performance over the past year and plan for the next financial year. It brings together policy-makers, and decisions are made there.	Brings policy-makers together to plan and assess the ministry's performance and plan for the following financial year.	Encourages stakeholder input in MoH’s achievements and participation in shaping the health agenda.	
At the national level, for the broader issues it is very much meetings followed by opportunities for input through written feedback. In the case of the Community Health Services Division, it is a smaller group of people, so it can be made to be intentionally more participatory. It is hard when you have the entire country trying to participate in one thing versus if you have stakeholders that are a bit more confined around a certain issue. I would say that overall, it’s been designed with the right level of participation, given the number of stakeholders that have to participate to develop large, overarching policies versus a division-specific policy, which allows more involvement.	Offers opportunities for input through written and participatory frameworks at the national and sub-national levels.	Encourages stakeholder input at the national and sub-national levels.	
The importance is that it leads to cooperative solutions, which often facilitates the understanding of decisions.	Leads to cooperative solutions, which often facilitates the understanding of decisions.	Comes up with cooperative solutions understandable to all stakeholders.	Permits joint identification of problems and possible solutions.
At the end of this activity, the major concerns are shared with all stakeholders in an environment where each party brings solutions	Major concerns are shared with all stakeholders and each party brings solution.	Allows joint identification of problems and possible solutions.	
The importance is to share the challenge we face in the health system to improve the health of the people that we care for. There are other sectors that work to improve the health of our people, and the importance of policy dialogue is to provide a mechanism so all these players can then contribute to improve the health of the population.	Permits sharing of challenges with all actors and allows all actors to contribute to solutions.	Allows joint identification of problems and possible solutions.	
From our experience it is a process that is very important because it allows everyone to be involved – the authorities, the health services and the population –in health policies.	Allows involvement of all stakeholders.	Allows involvement of all stakeholders.	Ensures a participatory approach to health planning, problem solving and validation of action plans.
PD brings together all stakeholders involved in the management of the health business, including those involved in the health committee on HIV/AIDS established by the Togolese State, the decentralised services at prefecture level other actors, politicians, traditional leaders in the prefecture, NGOs and all stakeholders who are represented in order to reflect on the health problem in our prefecture.	Brings together all stakeholders primarily involved in the management of health sector.	Ensures participation of all actors.	
This policy dialogue allows for the views of all stakeholders to be heard and for validating the action plans.	Allows for the views of all stakeholders to be heard and for validation of action plans.	Ensures participatory approach to health development.	
Through this dialogue the parties find solutions.	Enables parties to find solutions.	Ensures participatory problem solving.	

*MoH* ministry of health, *PD* policy dialogue

## Results

### Definition of health policy dialogue

We noted variations among the respondents in the definition of policy dialogue that were not necessarily linked to linguistic considerations or whether the policy dialogue took place at the national or sub-national level of the health system. There were variations with regard to the scope of policy dialogue, as summarised in Table 3. The definitions were grouped into five categories depending on whether they had an outcome, operational, process, forum or platform, or interactive and evidence-sharing orientation.

**Table 3:** Thematic definitions of policy dialogue

Definition of policy dialogue	Cape Verde	Chad		Guinea		Liberia		Togo	
	National	National	Sub-national	National	Sub-national	National	Sub-national	National	Sub-national
Outcome oriented policy dialogue	• Integrated approach to health questions and development of consensual strategic documents • Process to achieve mutual objectives jointly with partners	• A dynamic process involving all stakeholders through appropriate frameworks for exchange and consultation on ideas in the conduct of policy issues with the aim of having a consensus around a plan		• Exchanging ideas for the development of various strategic documents, and implementation arrangements and monitoring of programmes	• An approach that brings everybody together to solve health problems • Dialogue that binds all health workers to identify solutions to strengthen the health system			• Dialogue between stakeholders to achieve consensus on management of the health sector and on responsive, contextualised and implementable policies • Involvement of all stakeholders in discussions to find solutions, inform implementation and improve service delivery	• Dialogue among stakeholders to ensure harmonisation, seek commitment and find solutions to health problems
Policy dialogue as a tool (operational oriented policy dialogue)	• Tool to stop international partners from imposing their views and ideas	• Collaboration with partners on health policy						• Tool to strengthen cohesion in the implementation of sectoral policies by all stakeholders	
Process oriented definition		• Mechanism for the Ministry of Health to provide leadership • Reconciliation mechanism between donor and recipient		• Process that can set up a good health system for the benefit of the people	• Process for pooling interventions around a single pillar for guidance	• Series of consultative meetings • Coordination of stakeholders, ensuring participatory approaches for all stakeholders • Discussions of policy documents that help ministries of health to implement their strategic plans • Means to build the capacities of ministries of health in different areas • Mechanisms for stakeholders to provide technical input into health policies	• Series of consultative meetings		• Dialogue of stakeholders to discuss health policies
Forum/platform oriented policy dialogue			• A partnership among stakeholders	• Platform or mechanisms to ensure stakeholder input into policies and to pool resources to meet the health needs of populations • Forum that brings together different interest groups that are committed to addressing health issues					
Interactive knowledge-sharing policy dialogue				• A process of exchanging ideas and sharing evidence to inform policy development and implementation					

#### Outcome oriented policy dialogue

While the respondents recognised that policy dialogue was a process or an approach, they noted that it sought to achieve certain outcomes, implying that it aimed for certain objectives, for example the building of consensus on the strategic direction, as a donor respondent in Chad described it,

*A dialogue between stakeholders to achieve consensus on management of the health sector, consensus on contextualised and implementable policies.*

Some respondents defined policy dialogue as a process leading to the development of contextualised and responsive policies, such as a donor respondent from Togo, who said,

*A health policy dialogue is an exchange to achieve a policy that can cover, answer or solve the health problems of the population.*

The dialogue process was also said to seek to generate common goals and implementation arrangements, as stated by a MoH respondent in Guinea,

*A dialogue between stakeholders to achieve the definition of common goals and implement mechanisms and monitoring and evaluation of strategic frameworks in the health sector.*

In Cabo Verde, a respondent defined policy dialogue as a process leading to the achievement of mutual objectives.

#### Operational oriented policy dialogue

Some respondents defined policy dialogue as a tool that could be employed to address specific challenges, among which were guarding against external influence and ensuring stakeholder cohesion. An MoH respondent in Cabo Verde regarded policy dialogue as “*a tool to stop international partners from imposing their views and ideas*”, while for an MoH respondent in Togo said that, “*Health PD serves as a tool to strengthen cohesion in the implementation of sectoral policies by all stakeholders*”. In this regard, policy dialogue was perceived to be an operational tool to address alignment issues as well as to be used in the pursuit of common objectives.

#### Process oriented policy dialogue

To some respondents, policy dialogue was simply a process, implying that it could be an end in itself. Process oriented definitions associated policy dialogue with several dimensional elements, including meetings, as stated by a civil society organisation (CSO) respondent in Liberia, who defined policy dialogue as “*A series of consultative processes involving several stakeholders*”; a coordination tool, implying that policy dialogue serves to coordinate actors in the health sector; and a means to pool interventions, meaning that policy dialogue provides the opportunity to identify all the health interventions planned to be implemented. To some respondents, policy dialogue was a reconciliation mechanism that could be employed to bring together donors. This notion portrays policy dialogue as a process without envisaged outcomes.

#### Forum or platform oriented policy dialogue

Some respondents defined policy dialogue as a platform that could serve to address several issues, among which were resource mobilisation and gathering of actors around the table to discuss health issues, such as one MoH respondent in Guinea, “*This is a platform around which we can identify the most appropriate solutions*”. Similarly, a CSO respondent in Guinea defined policy dialogue as “*A platform where stakeholders discuss health strategies”.*

#### Interactive, knowledge-sharing and mutual learning oriented policy dialogue

To some respondents, policy dialogue was a mechanism that offered an opportunity to generate and share evidence. To a donor respondent in Liberia, “*It is a way of generating different ideas and perspectives from different organisations and people with different experiences*”. A respondent from the sub-national level in Guinea termed it as “*An exchange of information and sharing of approaches among the partners, providers and recipients.”*

There are others who perceived the process to be a mechanism to offer opportunities for capacity building, such as one MoH respondent in Liberia for whom it was “*A means to build the capacities of the ministries of health in the different areas*”.

### Importance of health policy dialogue

The themes that emerged regarding the perceived importance of health policy dialogue are summarised in Table 4. Policy dialogue was seen as important in (1) ensuring stakeholder participation, (2) improving harmonisation and alignment of actors, (3) supporting implementation of health policies, (4) fostering continued institutional learning, (5) providing a guiding framework, (6) stakeholder analysis, and (7) advocacy. There were no major differences in the perceived importance of policy dialogue between the Francophone, Lusophone and Anglophone countries or between the national and sub-national levels.

**Table 4:** Responses on the importance of health policy dialogue

Role of health policy dialogue	Cape Verde	Chad		Liberia		Guinea		Togo	
	National	National	Sub-national	National	Sub-national	National	Sub-national	National	Sub-national
Ensuring stakeholder participation		Joint identification of problems and possible solutions		• Ensures stakeholder participation in planning and monitoring • Facilitates collaborative and inclusive input into policy implementation at both the national and sub-national level				Joint identification of problems and possible solutions	• Joint identification of problems and possible solutions • Ensures participatory approaches to health planning, problem solving and validation of action plans
Improving harmonisation and alignment	• Achieving mutual objectives jointly with partners • Serves as a mechanism for consensus building	• Promotes alignment and harmonisation among actors		• To have good quality, consensual and MoH-owned plans that are supported by all stakeholders • Promotes alignment and harmonisation among actors		• Helps harmonise approaches, rationalise resources and improve efficiency of the health system • Improves coordination, alignment and harmonisation of actors and avoids duplications • Enables multi-sectoral coordination • Important in establishing a single framework for consultation, coordination, planning and monitoring of the implementation of health policies and strategies	• Promotes complementarity rather than competition among actors • Identifies population needs	• A mechanisms to enable seamless collaboration • Serves as a mechanism for consensus building	• Improves stakeholder collaboration and transparency
Supporting implementation of health polices	• To support implementation of national health plans	• To secure commitment to support implementation			• To secure commitment to support health programmes			• To mitigate implementation challenges • To support implementation of national health plans	
Continued institutional learning and evidence sharing			• Allows for contexualisation of evidence generated elsewhere	• Is an avenue for institutional learning • Serves as an information sharing forum		• Facilitates sharing of evidence among stakeholders		• Continued learning and information sharing	
Providing a guiding framework						• Gives guidance to countries on interventions			
Stakeholder analysis						• Serves to assess stakeholder positions, including external partners			
Advocacy		• Advocacy forum for resource mobilisation				• Powerful platform for advocacy on priority issues			

#### Ensuring stakeholder participation

The majority of the respondents were of the opinion that policy dialogue served as a mechanism for bringing stakeholders together to deliberate on health issues, providing an opportunity for all to contribute to health decision-making and planning and implementation of health programmes. Sub-national level respondents also added that within the policy dialogue, all actors contribute to problem identification as well as to possible solutions, as one sub-national respondent in Togo said, “*PD provides an opportunity to share challenges faced with all actors, also allows all actors to contribute to solutions*”.

Some respondents raised concerns regarding the ability of the policy dialogue process to ensure participation of stakeholders, given the numbers of actors involved in some of the processes.

*At the national level, for the broader issues it is very much sort of meetings, followed by limited opportunities for inputs through written feedback, because of the big numbers; but in the case of the Community Health Services Division, it’s a smaller group of people, so it can be more participatory.* (CSO respondent, Liberia)

Concerns were raised also that stakeholder participation was sub-optimal, noting that what sometimes was realised was stakeholder presence. This was partly attributed to the nature of the actors involved and their ability to synthesise, critique and discuss issues, as well as the untimely availability of relevant information, which prevented effective participation.

*There has been deficiency in participation. This relates to ensuring that participation is more than just representation but includes the means by which participants can methodically present, discuss, analyse, and select the best ideas to take forward, based on an informed decision process.* (Donor representative, Liberia)

Another constraint to effective participation was poor preparation. Stakeholders would go to meetings without prior knowledge of the issues to be discussed. The respondents emphasised the importance of adequate preparation for both the MoH and the other actors to get their ideas synthesised well.

*I think people come to the workshop and look at the agenda, but they don’t necessarily know the preparation you need before you attend. There is need to work with division heads and unit heads to help them understand how their roles can feed into a better outcome, in terms of preparation and participation, because those workshops often started at zero, and it would be great if those workshops started at like 50 %, with people having understood what they needed to have prepared and were ready, so that we can get to 100* % (MOH respondent, Guinea).

#### Improving harmonisation and alignment of actors

Another theme related to the role of policy dialogue in improvement of harmonisation of actors and alignment to country priorities as well as minimising duplication. The respondents noted that this was premised on the understanding that policy dialogue offers an opportunity to discuss investment priorities of stakeholders and government priorities, and that within this engagement consensus and commitment are realised.

*PD helps to harmonise approaches, rationalise resources and improve the efficiency of the health system.* (CSO respondent, Guinea)

*It’s very important, because without that dialogue, everyone will just get up and do his or her own thing, yet we have to conform to the system that is in place.* (CSO respondent, Liberia)

*Health policy dialogue is very important because in most cases people develop policies, buy those policies never get translated into strategic planning, never get operationalised. Policy dialogue, especially for us in Liberia, has helped us a lot to implement our policy and we have also actually gained a lot from the technical assistance attached to the policy dialogue so far.* (Donor respondent, Liberia)

In regard of harmonisation and alignment efforts, some respondents expressed concern over the MoH’s selective focus on certain issues or areas while leaving equally important issues out of the policy dialogue.

*PD needs to cover all areas; you shouldn’t concentrate on specific counties or policy issues. From what I really saw, more attention was given to some counties than others, and this is not right. The other thing I would say is, what was not part of the dialogue or what I don’t hear them talk about is health insurance.* (Donor respondent, Liberia)

#### Fostering continued institutional learning and evidence sharing

The respondents also identified evidence sharing and supporting institutional learning as areas that health policy dialogue could enhance. Specific mention was made of the need to ensure that all actors had the same information. Others felt that policy dialogue could be a forum to improve the use of evidence in policy-making and decision-making if the evidence was shared and discussed within the policy dialogue structures. Policy dialogue allows for bringing different pieces of evidence together; that is evidence from both formal (research, monitoring and evaluation, and surveys) and informal (experience and tacit knowledge) processes, given the fact that stakeholders come from different backgrounds.

*It helps the health system to generate innovative ideas and share best practices regarding the structure, processes and capacity of the health system.* (Donor respondent, Liberia)

*Policy dialogue allows us to have the same level of information and allows all actors to have the same vision. It also guards against the various stakeholders taking divergent decisions.* (MoH respondent, Togo)

*In its interactive character, PD promotes sharing of evidence among stakeholders.* (MoH respondent, Guinea)

Some respondents were of the view that policy dialogue facilitates stakeholder mapping and also serves as a platform for advocacy on investment priorities.

### Who coordinates health policy dialogue?

Respondents’ views were sought regarding who in their view coordinated the health policy dialogue at the national and sub-national levels. The majority of the respondents considered the MoH as the coordinating entity at the national level and the corresponding health department at the sub-national level. However, the MoH in some cases was judged to be authoritarian in its coordination role.

*When we are in the forums, the MoH just imposes its ideas. This approach has led to some weaknesses and some things are not enforced. For example, we asked for the construction of certain infrastructure and we were told that it would possible. But until now this has not happened. We are in 2015.* (Sub-national district health office respondent, Chad)

Some respondents in Togo and Liberia reported that the MoH and WHO jointly coordinated the policy dialogue process at the national level, while in Chad some respondents named WHO as having that role. Noteworthy is the fact that some respondents in Guinea stated that they did not know who coordinated the process.

Some respondents expressed reservations regarding the manner in which the MoH conducted the policy dialogue, specifically the decision of making policy dialogue the responsibility of only one of its departments. The concern stems from the fact that the issues covered in the policy dialogue process are vast, and thus there is need to have capacity built in all MoH departments for policy dialogue.

*We need to have more people in the ministry to take part in and own and manage components of the PD. Currently, it is only the Policy and Planning Department that understands policy and planning and people just say, ‘that’s that division over there that deals with that’. Let’s be able to say, ‘That’s my division too and here’s how I need to feed into that process and play a role’.* (CSO respondent, Liberia)

The selective involvement of only one department in the policy dialogue process was reported to also compromise MoH ownership of the policy dialogue programme at the higher level, given that the senior officers were not known to be very participative, which would impact the realisation of alignment efforts.

*There are problems with ownership and lack of leadership by the MoH. This is because the PD is not carried high enough. Very few managers are aware of the health policy dialogue.* (Donor respondent, Chad)

Other constraints noted included the weak coordination of the policy dialogue efforts between national and sub-national levels stemming partly from the weak capacity at the sub-national level; the top-down approach to health planning; and the divergence of views among the actors at the national level in some of the areas regarding the best policy option.

*There is very little involvement of the county health teams in planning. If you really want to get them involved, you should have started the dialogue with them right at the beginning, to take their inputs with you into planning. Over here, there is a top-down approach with most of the disease programmes. Of course, there are disagreements and differences of opinion, but then, that’s what democracy is all about; that is why we have dialogue.* (Sub-national CSO respondent, Chad)

## Discussion

Our study identified the diverse definitions and variations in perceived importance of policy dialogue among the respondents, differences that were influenced by neither linguistic considerations nor the level of the health systems where the dialogue took place. The respondents also noted the importance of good coordination in the policy dialogue process, as well as the weaknesses that compromise the process.

### Definition of policy dialogue

The literature highlights the fact that policy dialogue means different things in different countries, partly attributed to the official language of the country [4], but that was not the case in our study. The lack of this difference may be explained by the fact that the countries we studied had been implementing a policy dialogue capacity building programme over a three-year period through which a common understanding of the concept had been achieved to some extent. This then may imply that the persisting differences arose from individual experiences not linguistic considerations or level of the health system where the dialogue took place.

While some respondents defined policy dialogue as a process others regarded it as was a tool or a means to achieve certain objectives. The literature highlights variations in the definition, with some scholars arguing that the varied interpretation of the concept may impact the outcomes from the process [12]. For example, viewing policy dialogue as a capacity-building process or a tool to bar external influence might mean missing the opportunity to realise the outcomes the process engenders with regard to consensus building, ownership of the outcomes and commitment by all actors. This would also influence how the process is conducted, given that the facilitation mode for a capacity building process would differ from that for a policy dialogue process. In addition, partner expectations would vary, as they are premised on how the process is conceived.

### Importance of health policy dialogue

To the majority of the respondents, policy dialogue served as a mechanism for stakeholders to come together to deliberate on health issues, providing them the opportunity to contribute to decision-making, planning and implementation of programmes. The literature emphasises the need for interaction among stakeholders at the different stages of policy development to encourage exchange of knowledge and experience in order to have the best possible outcomes [13]. Some scholars recommend that the policy dialogue be well structured so that all the stakeholders have a chance to contribute [6]. Rajan et al. [4] state that policy dialogue can lead to key policy decisions with the buy-in and ownership of a wide range of stakeholders. This is crucial, because policy implementation success is directly dependent on the buy-in of the stakeholders who are involved in the implementation process.

While it is clear that stakeholder involvement is an important component of the policy dialogue process, caution should be exercised to ensure that only a manageable number of participants are involved, for meaningful dialogue. That some groups were too large was a concern raised by some respondents in our study. Some scholars consider a small number of participants with a focused purpose to be the most effective in the policy dialogue process [6]. On the opposite end of the spectrum, some respondents highlighted the fact that participation was not enough. The bottom line is that a good policy dialogue process must be able to guarantee a good level of participation without compromising the process by having too few or too many participants, as either would have sub-optimal outcomes. An option is to have thematic groups addressing specific policy issues, which then feed into a sectoral strategic policy dialogue structure for final decision-making. The involvement of the sub-national level is crucial, given the fact that it has on-site experience and can help find the common ground, especially taking into consideration that contextual issues may impact policy implementation.

Our study found that stakeholder participation was limited owing to poor advance preparation, as many of the actors attended the meetings without proper knowledge of the issue at stake. The literature emphasises the importance of a good preparatory process. For example, sharing evidence briefs ahead of the meeting can go a long way in helping stakeholders synthesise information relevant to the policy, including using local data and studies to describe a problem and the options for addressing it and their implementation considerations [14]. Rosen [15] states that sending out surveys and information materials stimulates reflection and development of some form of opinion, which is a prerequisite for participation in rather complex discussions about setting priorities in health care. Providing meeting materials early would go a long way in ensuring that stakeholders have the relevant tools to engage in and influence the policy dialogue process.

Improving actor harmonisation and alignment was another perceived important role of policy dialogue. This view has been highlighted by other scholars as well. For example, Carcasson [16] states that feedback from participants in some dialogues indicated that they left the meeting with a sense of enhanced mutual understanding of each other’s position, paving way for pursuing of a common vision. Literature highlights the benefits of a well conducted policy dialogue as (1) assisting the key actors to see the problems from each other’s perspective, improving understanding of the impact that policies and programmes can have on various groups [17]; (3) fostering understanding of the common solutions with which to move forward [18]; and stimulating debate on and facilitating understanding of complex issues while encouraging consensus around various health priorities [19]. We emphasise the fact that realising actor harmonisation and alignment through policy dialogue calls for good preparation and facilitation of the process.

Continued institutional learning and evidence sharing also were cited as areas that could be enhanced by policy dialogue. The value of these two elements cannot be overstated, as the use of evidence helps stakeholders to develop evidence-informed policies, as well as allowing their continued learning and improvement. Lavis et al. [20] indicate that describing the features of a problem in a policy brief using evidence can be particularly important for highly politicised topics where the very nature of the problem is contentious.

### Coordination of health policy dialogue

In our study the question as to who was responsible for coordination of the policy dialogue simply sought to find out who had the duty to oversee the policy dialogue process and to some extent who took ownership of the policy dialogue process. Sundwall et al. [21] define ownership as the situation when the government decides the direction and content of the development process after engaging in discussions with major stakeholders. It makes sense that many respondents associated the policy dialogue coordination responsibility with the MoH, a government body. That some of the respondents in Guinea did not know who coordinated the policy dialogue process may be explained by the fact that participation of the actors from the different agencies in meetings was not consistent and that the meetings were chaired by different individuals each time, or that the respondents had been in only a few policy dialogue processes.

Although the majority of the respondents in this study were keen to point out that the MoH was responsible for coordinating the policy dialogue process, some of them strongly expressed the view that the MoH just imposed its agenda. Such an approach by the MoH would hamper cooperation and, subsequently, the alignment and harmonisation of the key stakeholders. In addition, we emphasise the fact that MoH leadership of the dialogue process is an institutional issue not a prerequisite for government departments selected as stakeholders in the dialogue process. In our study, the respondents pointed to the selective involvement of MoH officials despite the fact that the issues addressed were vast. This may perhaps explain the occurrence of what was described by some of the respondents as selective attention to issues while ignoring others that were equally important. Inadequacy of the capacity to coordinate the policy dialogue at the sub-national level was reported, as was the related top-down approach to policy dialogue adopted by the MoH. The use of the traditional top-down approach to policy-making means that many of policies are rarely implemented per their set objectives [22]. Indeed, some of the respondents in our study had opposing views on whether national level or sub-national policy options were better.

There are limitations to our study. We acknowledge that the policy dialogue may be modified given the policy issue under consideration and as such, may be perceived differently. We however did not assess perspectives of stakeholders for related policy issues and this in itself may have impacted how actors defined policy dialogue. The strength however is the assessment of views across different countries and at the national and subnational level. This provides an opportunity to synthesise findings that can be applied in several low income countries to improve policy dialogue processes.

## Conclusion

Policy dialogue offers several opportunities to improve stakeholder participation in policy development and implementation and to improve aid effectiveness. However, there is need for conceptual clarity in order to ensure pursuance of common objectives. While it is clear that stakeholder involvement is an important component of the policy dialogue process, caution should be exercised to involve a manageable number of participants, for meaningful dialogue. Ownership and coordination of the policy dialogue process are important aspects. The institutional capacity of the MoH for coordinating the policy dialogue process needs to be built as opposed to strengthening selected departments within the MoH. Likewise, the capacity for policy dialogue needs to be built at the sub-national level, alongside fostering a bottom-up approach in policy processes.

## Abbreviations

CSO, civil society organisation; MoH, ministry of health; PD, policy dialogue; WHO, World Health Organization
